# Probability Weighting Functions Derived from Hyperbolic Time Discounting: Psychophysical Models and Their Individual Level Testing

**DOI:** 10.3389/fpsyg.2016.00778

**Published:** 2016-05-26

**Authors:** Kazuhisa Takemura, Hajime Murakami

**Affiliations:** ^1^Institute of Decision Research, Waseda UniversityTokyo, Japan; ^2^Department of Psychology, Waseda UniversityTokyo, Japan

**Keywords:** probability weighting function, hyperbolic discounting, prospect theory, decision under risk, probability judgment

## Abstract

A probability weighting function (*w*(*p*)) is considered to be a nonlinear function of probability (*p*) in behavioral decision theory. This study proposes a psychophysical model of probability weighting functions derived from a hyperbolic time discounting model and a geometric distribution. The aim of the study is to show probability weighting functions from the point of view of waiting time for a decision maker. Since the expected value of a geometrically distributed random variable *X* is 1/*p*, we formulized the probability weighting function of the expected value model for hyperbolic time discounting as *w*(*p*) = (1 − *k* log *p*)^−1^. Moreover, the probability weighting function is derived from Loewenstein and Prelec's (1992) generalized hyperbolic time discounting model. The latter model is proved to be equivalent to the hyperbolic-logarithmic weighting function considered by Prelec (1998) and Luce (2001). In this study, we derive a model from the generalized hyperbolic time discounting model assuming Fechner's (1860) psychophysical law of time and a geometric distribution of trials. In addition, we develop median models of hyperbolic time discounting and generalized hyperbolic time discounting. To illustrate the fitness of each model, a psychological experiment was conducted to assess the probability weighting and value functions at the level of the individual participant. The participants were 50 university students. The results of individual analysis indicated that the expected value model of generalized hyperbolic discounting fitted better than previous probability weighting decision-making models. The theoretical implications of this finding are discussed.

## Introduction

Probability weighting functions (*w*(*p*)) are widely known in behavioral decision theory and behavioral economics. A probability weighting function is considered to be a nonlinear function of probability (*p*). There are several probability weighting decision-making models for representing probability weighting functions (e.g., Tversky and Kahneman, 1992; Prelec, 1998; Gonzalez and Wu, 1999; Takahashi, 2011; Zhang and Maloney, 2012; Takemura, 2014). However, most of the proposed models are not related to traditional psychological theories such as psychophysics and learning theory, with the exception of studies by Prelec and Loewenstein (1991), Tversky and Kahneman (1992), Kusev et al. (2009), and Takahashi (2011). Prelec and Loewenstein (1991) first pointed out that there are common properties between risky and intertemporal choices from an axiomatic point of view. They illustrated that the choice patterns of risky and intertemporal choices are very similar in terms of their axiomatic properties. From this point, they suggested that there are some common behavioral foundations between the probability weighting function and the time discounting function. Importantly, they illustrated some common axiomatic properties between the probability weighting function and the time discounting function, although no psychological account was given.

In prospect theory (proposed by Kahneman and Tversky, 1979), the probability weighting function has psychophysical foundation as well as the value function. Additionally, in cumulative prospect theory (proposed by Tversky and Kahneman, 1992), the probability weighting model parameter represents “probability discriminability” and “diminishing sensitivity”—assumptions derived from psychophysical research. Furthermore, the probability-weighting model in Kusev et al. (2009) accommodates a memory parameter (accessibility to events from memory). Their results revealed evidence that exaggerated risk is caused by the accessibility of events in memory; in other words, the weighting function varies as a function of the accessibility of events. This suggests that people's experiences of events leak into decisions even when risk information is explicitly provided. In addition, Takahashi (2011) combined psychophysical theory with Cajueiro's (2006) q-exponential function for explaining time discounting, proposed a new general model, and then derived Prelec's (1998) probability weighting function as a special case. The merit of combining a probability weighting model with a time discounting model is to create an integrated human decision model. The time discounting model and decision under risk, whose probability distribution is known, are both important areas in behavioral decision research. However, there seems to be a strong connection between them.

The purpose of the present study is to show several probability weighting functions from the viewpoint of a decision maker's waiting time to receive an outcome. The study proposes another type of probability weighting function derived from the hyperbolic time discounting model in a simpler form. We assume only the hyperbolic time discounting function and Fechner's (1860) logarithmic psychophysical function. By the assumptions of the geometric distribution of waiting time and Fechner's (1860) law, we provide a new account of Prelec's (1998) probability weighting function.

Last, we perform an experimental study to illustrate the fitness of our models and previous models. Concerning the empirical research on probability functions, important research has been conducted by Stott (2006). In particular, Stott (2006) reviewed eight different forms of the probability weighting function (linear model, power model, log-odds model, Tversky–Kahneman model, Wu–Gonzalez model, two versions of Prelec's model, and non-parametric model) and reported parameters estimated from multiple empirical papers over a period of 10 years. He also reported an extensive empirical study for 96 participants by utilizing 90 different gamble stimuli. His study compared fits on a total of 256 combinations of cumulative prospect theory functional forms, including eight probability functions, eight value function forms, and four choice functions. Based on this study, he concluded that the best model has a risky weighting function of the simple version of Prelec's (1998) model, a power value function, and a logit choice function. We also examined the Prelec (1998) model using the power function for a value function by comparing the proposed model and some previous models. Although the number of participants was limited (total of 50 participants), Prelec's (1998) general version of the probability weighting model fitted our data better than the other models did. This finding shows that the best model is the probability weighting function based on Loewenstein and Prelec's (1992) generalized hyperbolic time discounting model.

## Probability weighting function based on prospect theory

Nonlinear utility theory was proposed to explain several anomalies of expected utility theory, such as the Allais paradox (Allais, 1953). This body of theory is a generalization of expected utility theory (Tamura et al., 1997; Starmer, 2000). This theory is called the nonlinear utility theory (Fishburn, 1988; Edwards, 1992) or generalized expected utility theory (Quiggin, 1993) in the field of economics, although it is mathematically equivalent to the theory of non-Lebesgue integration in fuzzy measure theory in the field of engineering (Sugeno and Murofushi, 1993; Takemura, 2014). Nonlinear utility theory often assumes a non-additive probability weighting function that converts probabilities for which additivity does not hold, even if probability information is given for decision making under risk, such as in the case of the Allais paradox. A non-additive probability is sometimes referred to as a “capacity,” but in some cases (e.g., in the field of engineering) is called a “fuzzy measure.” Its mathematical definition is the same despite these varying names. A non-additive probability refers to a set function, *w*: 2^Ω^ → [0, 1] from an aggregate consisting of subsets of a nonempty set, Ω, to a closed interval, [0,1], which is also a set function that satisfies both a boundedness condition (*w*(ϕ) = 0, *w*(Ω) = 1) and a monotonicity condition (if the relation of subsets E and F of Ω is E ⊆ F, then the relation *w*(E) ≦ *w*(F) is satisfied). A non-additive probability is so named because it does not necessarily satisfy the conditions of additivity.

**Figure 1:**
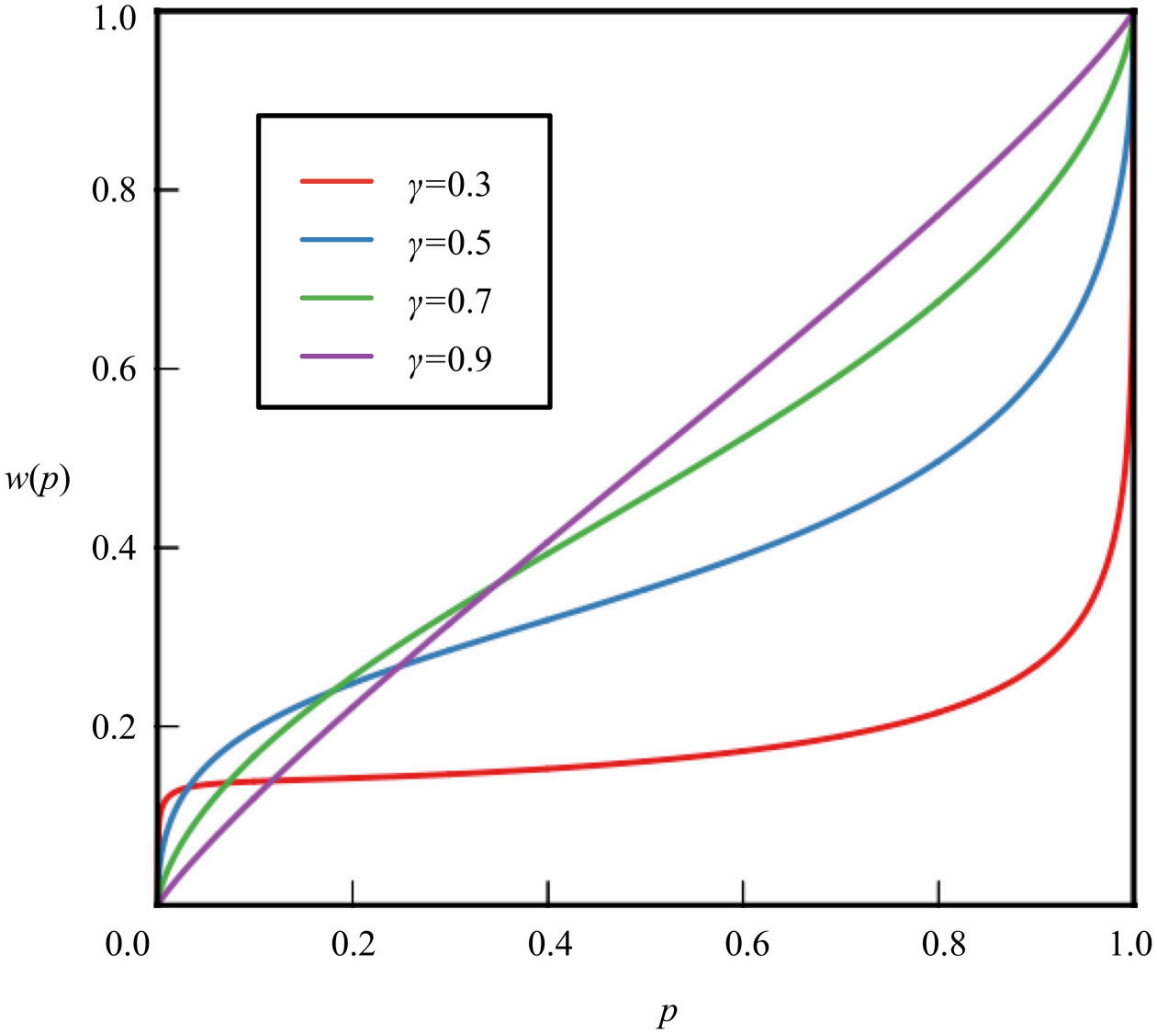
**Probability weighting function based on the study of Tversky and Kahneman (1992)**.

**Figure 2:**
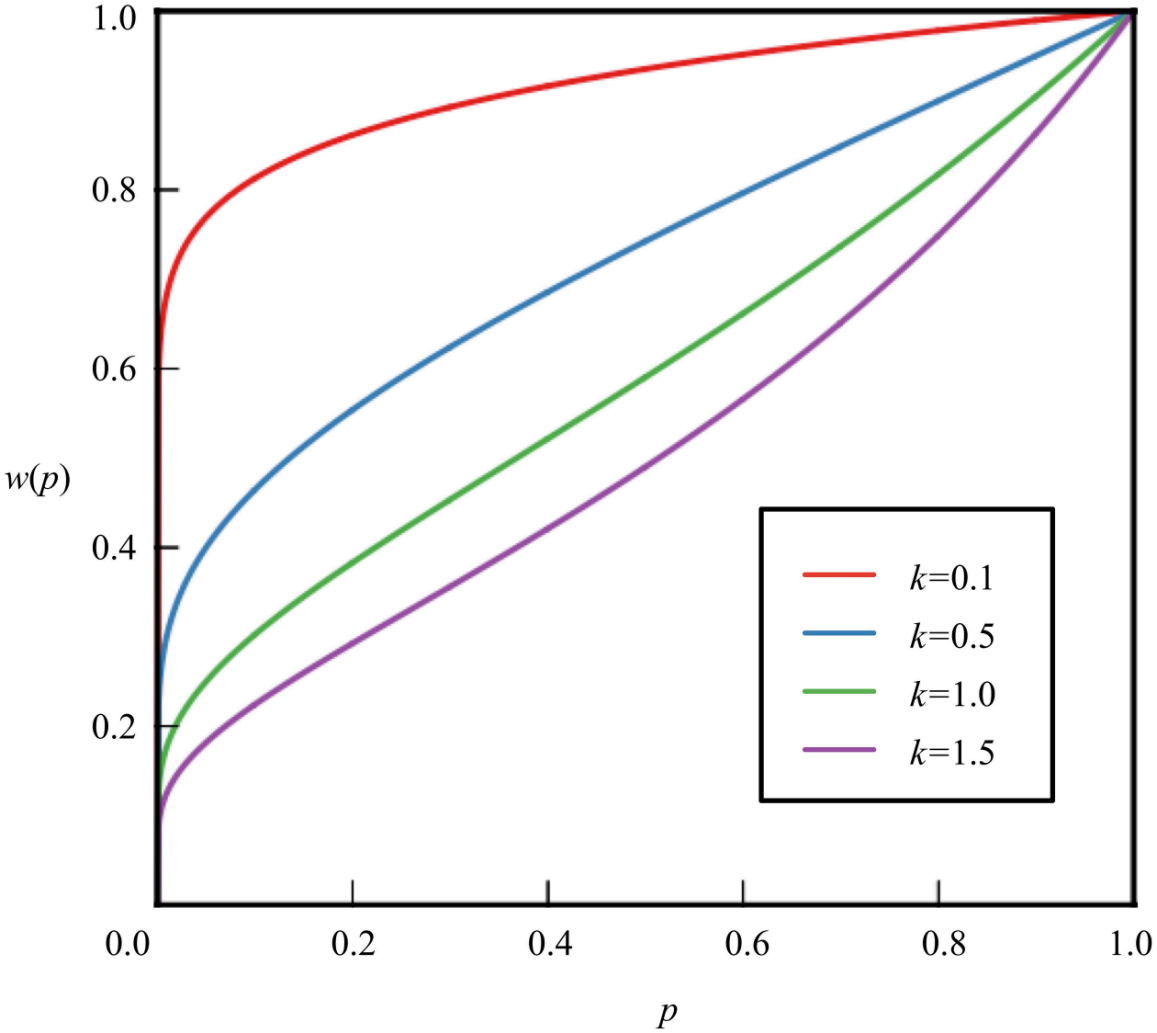
**Probability weighting function derived from hyperbolic discounting model**.

Moreover, a non-additive probability weighting function in prospect theory has the following properties: *w*(0) = 0 and *w*(1) = 1; it is of the form shown in Figures 1, 2. Assuming that the probability weighting function is *w* and that the probability is *p*, the probability weighting function has the following qualitative characteristics.

- It satisfies the condition of *w*(*p*) + *w*(1 − *p*) ≤ 1.
- It overvalues the probability when the probability is very low, engendering the relation of *w*(*p*) > *p*.
- It shows non-proportionality—i.e., $\frac{\text{w}\left(\text{pq}\right)}{\text{w}\left(\text{p}\right)}\leq \frac{\text{w}\left(\text{pqr}\right)}{\text{w}\left(\text{pr}\right)}$.
- It has non-continuity near the endpoints.

Tversky and Kahneman (1992) conducted an experiment to obtain detailed information about the value and weighting functions. They recruited a total of 25 graduate students at Stanford and Berkeley. The experiment was conducted on a computer, which computer displayed a prospect and its expected value. For example, the probability of gaining 150 dollars was 25% and the probability of gaining 50 dollars was 75%. The display also included a descending series of seven sure outcomes logarithmically spaced between each of the seven sure outcomes and the risky prospect (Tversky and Kahneman, 1992). They assumed the following power functions as value functions.

$$\begin{array}{lll} v\left(x\right) & = & x^{\alpha }\left(\text{when }x\geq 0\right)\\ v\left(x\right) & = & {-\lambda \left(-x\right)}^{\beta }\left(\text{when }x<0\right) \end{array}$$

Based on the results of the selections in this experiment, they performed a nonlinear regression analysis and estimated 0.88 for both α and β, and 2.55 for λ. The fact that the estimated values of α and β are less than one indicates that the value function is concave downward in both the areas of gain and loss. The estimated value of λ suggests that loss has an impact that is approximately twice as great as that of profit, implying substantial strength for loss aversion.

They further considered the following functions as specific decision weight functions, *W*^+^ and *W*^−^, of cumulative prospect theory, and estimated the form of the decision weight functions illustrated in Figure 1 from this selection experiment.

(1) $$\begin{array}{l} W^{+}\left(p\right)=\frac{p^{\gamma }}{{\left(p^{\gamma }+{\left(1-p\right)}^{\gamma }\right)}^{\frac{1}{\gamma }}},W^{-}\left(p\right)=\frac{p^{\delta }}{{\left(p^{\delta }+{\left(1-p\right)}^{\delta }\right)}^{\frac{1}{\delta }}}. \end{array}$$

Another well-known two-parameter model was proposed by Prelec (1998). The functional form is as follows:

(2) $$\begin{array}{l} w\left(p\right)=\text{exp}\left[{-\delta \left(-\text{ln p}\right)}^{a}\right], \end{array}$$

where $0<\alpha <1;\;w\left(0\right)=0;\;w\left(\frac{1}{e}\right)=\frac{1}{e};\;w\left(1\right)=1$.

In most empirical studies of probability weighting functions, assuming δ equals 1, a one-parameter model is used, which can be described as follows:

(3) $$\begin{array}{l} w\left(p\right)=\text{exp}\left[{-\left(-\text{ln p}\right)}^{a}\right]. \end{array}$$

## Probability weighting function derived from hyperbolic time discounting

Hyperbolic discounting is a mathematical model devised as an improvement over the exponential discounting model, a time-consistent model of discounting. Hyperbolic discounting can be described as follows:

(4) $$\begin{array}{l} f\left(D\right)=\frac{1}{1+kD}, \end{array}$$

where *f* (*D*) is the discount factor that multiplies the value of the reward, *D* is the delay in the reward, and *k* is a parameter governing the degree of discounting. In this study, we derive the logarithmic hyperbolic probability weighting function from *f* (*D*), assuming that the trial is a geometrically distributed random variable and that the delay is evaluated using Fechner's (1860) law. Similar assumptions were previously adopted in Takahashi's (2011) study, in which he considered a link between time discounting and Prelec's (1998) two-parameter probability weighting function. Takahashi (2011) derived Prelec's (1998) probability weighting function Equation (2) from a q-exponential time discount function (Cajueiro, 2006). In this study, however, we adopt a more direct assumption of time discounting and then derive the different probability weighting function of Equation (2). Interestingly, we derive a hyperbolic probability weighting function that has been derived from a different theoretical foundation of probability weighting function in Equation (2) from a generalized time discounting model (Loewenstein and Prelec, 1992).

We assume that the probability weighting function, *w*(*p*), is psychologically related to the delay discounting function, *f* (*D*). The key assumption in this is equating delay, *D*, with the expected number of Bernoulli trials to obtain one success (1/*p*), and considering that the perceived delay is a logarithmic function of the delay based on Fechner's (1860) logarithmic psychological function. Some of the previous empirical studies on time discounting (Rachlin et al., 1991, 2000) indicated that the odds of receiving probabilistic gain ((1-*p*)/*p*)) can be considered to be the delay, *D*, in Equation (4). Rachlin et al. (1991, 2000) suggested that the probability discounting has the same psychological foundation as that of time discounting.

Because the odds of receiving probabilistic gain ((1-*p*)/*p*)) is equal to the expected number of Bernoulli trials to obtain one success (1/*p*) − 1 (i.e., (1/*p*) −1), we have a similar assumption by Rachlin et al. (1991, 2000). However, we also assume that the perception of waiting time holds Fechner's logarithmic psychophysical law as hypothesized by Takahashi (2011).

Let *X* be the number of Bernoulli trials required to obtain one success, supported on the set {1, 2, 3, … }. This is the probability that the first occurrence of success requires *k* independent trials, each with probability *p* of success. If the probability of success on each trial is *p*, then the probability that the *k*th trial is the first success is:

(5) $$\begin{array}{l} P\left(x=k\right)={\left(1-p\right)}^{k-1}p\text{ for }k=1,2,3,\ldots \end{array}$$

The probabilities form a geometric sequence. The expected value of a geometrically distributed random variable *X* is 1/*p*, and the variance is (1 − *p*) ∕ *p*^2^.

Assuming Fechner's (1860) law, the delay can be considered to be a logarithmic function of the number of trials—that is, *D* = *ln*(1∕*p*) = −*ln*(*p*).

The probability weighting function derived from hyperbolic time discounting (Figure 2) is:

(6) $$\begin{array}{l} w\left(p\right)=\frac{1}{1-k\text{ ln }p}, \end{array}$$

where *p* is the probability, *k* is a constant, and *k* > 0.

The indicator −ln *p* (=ln (1∕*p*)) is also related to the median of trials to some extent. That is, the median of the trials is:

(7) $$\begin{array}{l} Median\left(X\right)=\frac{-1}{{\text{log}}_{2}\left(1-p\right)}. \end{array}$$

Since the geometric distribution is skewed, −log *p* is considered to be an approximation of the median of trials. On this interpretation, the probability of the weighting function is:

(8) $$\begin{array}{l} w\left(p\right)=\frac{1}{1-k\left(\frac{1}{{\text{log}}_{2}\left(1-p\right)}\right)}, \end{array}$$

where *p* is probability, *k* is a constant, and *k* > 0.

Although the median model is related to the hyperbolic model, the median model in Equation (8) will be discussed elsewhere in more detail.

## Probability weighting function derived from generalized hyperbolic time discounting

The probability weighting function is also derived from Loewenstein and Prelec's (1992) generalized hyperbolic time discounting model.

Their model is as follows:

(9) $$\begin{array}{l} f\left(D\right)={\left(1+\alpha D\right)}^{-\gamma ∕\alpha }. \end{array}$$

Letting α = *k* and γ/α = β, their model can be written as follows:

(10) $$\begin{array}{l} f\left(D\right)={\left(1+kD\right)}^{-\beta }. \end{array}$$

Assuming Fechner's (1860) law, the delay can be considered to be a logarithmic function of the number of trials—that is, *D* = ln(1 ∕ p) = −ln(p).

The probability weighting function derived from the generalized hyperbolic time discounting Equation (6) is as follows:

(11) $$\begin{array}{l} w\left(p\right)={\left(1-k\text{ ln }\left(p\right)\right)}^{\beta }, \end{array}$$

where *p* is probability, *k* is a positive constant, and β is a negative constant.

The model (11) is the same as the hyperbolic-logarithmic weighting function considered by Prelec (1998) and Luce (2001).

We also proposed a new psychophysical model based on generalized hyperbolic time discounting. Since the geometric distribution is skewed, as noted previously, −log *p* is considered to be an approximation of the median of trials. On this interpretation, the probability of the weighting function is as follows:

(12) $$\begin{array}{l} w\left(p\right)={\left(1-k\left(\frac{1}{{\text{log}}_{2}\left(1-p\right)}\right)\right)}^{\beta }, \end{array}$$

where *p* is probability, *k* is a positive constant, and β is a negative parameter.

## Method of psychological experiment

We adopted experimental methods similar to those of previous studies (Tversky and Kahneman, 1992; Gonzalez and Wu, 1999). Although the number of participants was limited, it appears to have been sufficient for illustrating model comparison. A psychological experiment was conducted to estimate a probability weighting function whose stimuli were presented graphically.

### Participants

We report data for 50 participants (35 females and 15 males, aged 19–24 years). All participants were undergraduate students in psychology. They were paid 2000 Japanese yen (about 20 dollars) for participating in four sessions that lasted 1 h and 30 min each.

### Materials

The basic design consisted of 15 two-outcome wagers with 11 levels of probability associated with the maximum outcome, in the same manner of the study by Gonzalez and Wu (1999). The two outcome wagers were (in yen), 2500-0, 5000-0, 7500-0, 10,000-0, 15,000-0, 20,000-0, 40,000-0, 80,000-0, 5000-2500, 7500-5000, 10,000-5000, 15,000-5000, 15,000-10,000, 20,000-10,000, and 20,000-15,000. Note that all wagers offered nonnegative outcomes, and that prospect theory codes all such outcomes as gains. The 11 probability levels were 0.01, 0.05, 0.10, 0.25, 0.40, 0.50, 0.60, 0.75, 0.90, 0.95, and 0.99. Nine of these wagers (randomly chosen) were repeated to provide a measure of reliability. Thus, we used total of 174 materials (165 and 9 materials). Except for the restriction that the same wager could not appear in two consecutive trials, the repeated wagers were randomly interspersed within the complete set of wagers. The outcomes and probabilities presented to the participants are shown in the Supplementary Material.

### Procedure

**Figure 3:**
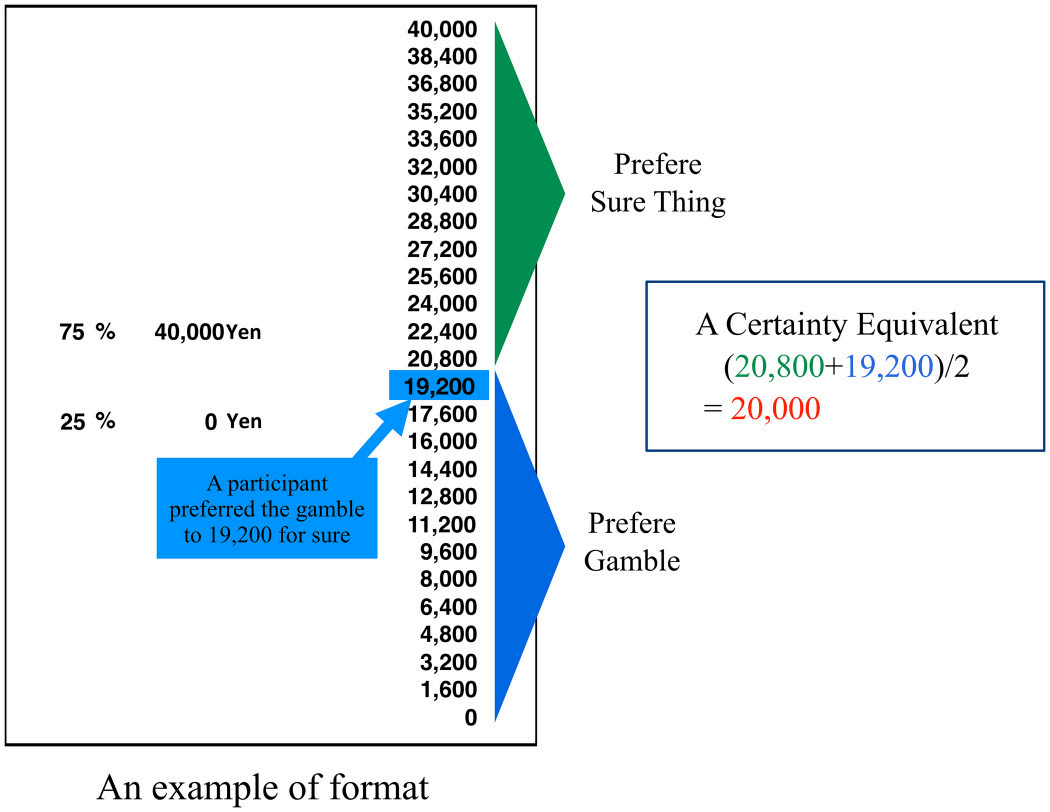
**An example of presented format in the experiment**.

A simplified computer program following the procedure outlined in Tversky and Kahneman (1992) and Gonzalez and Wu (1999) was used in this study. The program presented one wager on the screen and asked the participants to report a certainty-equivalent value (yen) from a menu of possibilities. Tversky and Kahneman (1992) used logarithmically spaced distributions of certainty between the minimum and the maximum of the prospect. However, the current study used linear distributions of certainty/sure outcomes, as in Gonzalez and Wu's (1999) study, because of the simplicity of the manipulation involved. The screen for this particular wager offered the participant a choice of certainty-equivalent values. The certainty equivalents (or cash equivalents) were determined by the midpoint between the lowest accepted and highest rejected values, as in the study of Tversky and Kahneman (1992) and Gonzalez and Wu (1999). This method of direct reporting of the certainty-equivalent value was a simplified version of the method used in the study of Tversky and Kahneman (1992) and Gonzalez and Wu (1999). The format presented in the experiment is shown in Figure 3. The participant reported the certainty-equivalent value. Once the certainty equivalent was determined within a range, a second screen with a new menu list was presented. The program presented wagers to each participant in random order.

## Results and discussion of the experiment

Reliability for the nine repeated wagers was measured by intraclass correlation. The median of 50 intraclass correlations computed for the individual subject data was 0.98, with a range of 0.36–1.00. The experimental procedure appears to have elicited relatively high levels of reliability for most participants. The median of the reliability was slightly higher than that for the findings of Gonzalez and Wu (1999). However, 4 out of 50 intraclass correlations were below 0.70 (the lowest being 0.36, the second lowest being 0.57, the third lowest being 0.63, and the fourth lowest being 0.69). Therefore, we omitted these data for further analysis, and the 46 remaining data were analyzed.

As suggested by Gonzalez and Wu (1999), estimation of the probability weighting function in the context of nonlinear utility theory presents challenging problems. We used the nonparametric estimation algorithm in the same manner as the study of Gonzalez and Wu (1999), though monotonicity on *v* and *w* was implemented into the estimation procedure. Using the certainty equivalents from 165 two-outcome gambles, pick the starting values for 11 *w*()s (i.e., one for each *p*) and eight *v*()s (i.e., one for each money amount). The algorithm proceeds as follows, with the superscript denoting the *i*th iteration (Gonzalez and Wu, 1999):

1. Interpolate for vi(CE): using the estimates of v() for the current iteration, which are based on the eight stimuli money amounts, interpolate to find vi(CE) for each of the 165 certainty equivalents; these 165 vi(CE) values will be used as “data” for the estimation in Steps 2 and 3.
2. Fix all v() values to the current iteration values and estimate the eleven wi values using an iteratively reweighted, nonlinear least-squares algorithm.
3. Fix the 11 w() values to the current iteration values and estimate the eight vi() values using an iteratively reweighted, nonlinear least-squares algorithm.
4. If an optimum value is found, then stop; otherwise, increment iteration counter i and repeat.

We first computed the value function parameter assuming the power function using the abovementioned nonlinear least-squares algorithm for the median values of certainty equivalents for all participants. The estimated value of power was 0.80. We then computed the power value for each participant. The median power value was 0.85 (the lowest was 0.34 and the highest was 1.00). The power value in the study by Tversky and Kahneman (1992) was 0.88, whereas the median value in the present study was 0.80, considering median values of certainty to be equivalent for all participants. The value of α in this study is similar to that in the original study by Tversky and Kahneman (1992). This finding suggests that the experiment replicated the original research of prospect theory.

It should be noted that the value function is convex for the loss domain, as indicated by Tversky and Kahneman (1992). As Stott (2006) demonstrated in his review, the alpha (power value in gain domain) and beta (power value in loss domain) parameters reported by Tversky and Kahneman (1992) are clear outliers when compared to parameter values reported since then, which include two papers (Wu and Gonzalez, 1996; Gonzalez and Wu, 1999) that report parameters very close to 0.50. Stott (2006) reported a value of 0.19 for alpha. As Stott (2006) pointed out, these values may be related to the size of the payoffs used in the gamble stimuli.

To examine whether the finding in that study was replicated or not, we compared the value of a parameter of this study to the corresponding value in the previous study. The value of γ in the study of Tversky and Kahneman (1992) was 0.61, whereas the value of γ in the present study was 0.63 using the median values of the certainty equivalent for all participants. The value of γ in this study is similar to that in a previous study by Tversky and Kahneman (1992). This finding suggests that the experiment replicated the results of the previous study. Stott (2006) reported the values of γ for seven studies reviewed between 1992 and 2006. According to his review, the value of γ was in the range of 0.50 to 0.96, and the median was 0.61. The value of γ in this study is also close to the median value in a previous study reviewed by Stott (2006).

We fitted the individual choice data with not only the probability weighting function proposed by Tversky and Kahneman (1992), but also with the probability weighting functions of a simple version of the Prelec model (1998) and our derived models.

**Figure 4:**
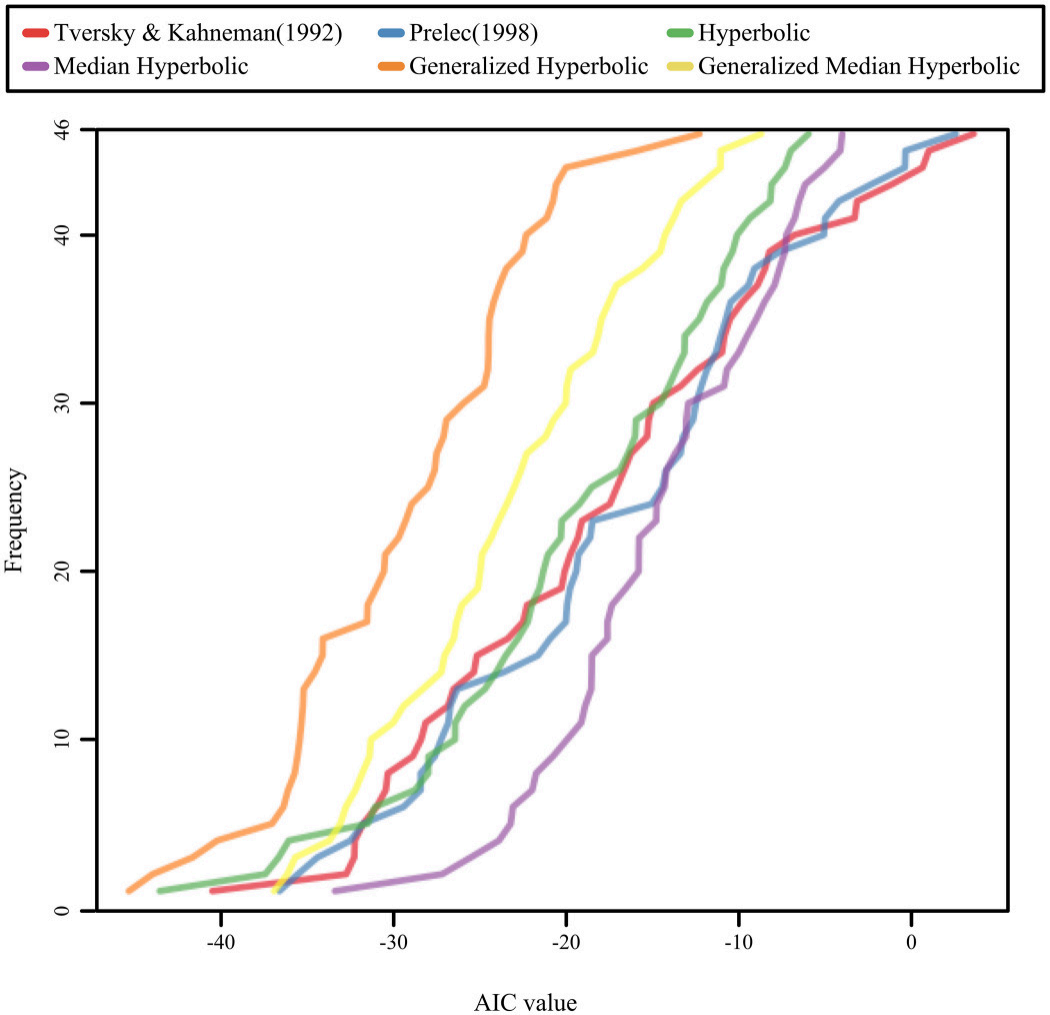
**The cumulative distributions of the AIC values for the six models**.

**Figure 5:**
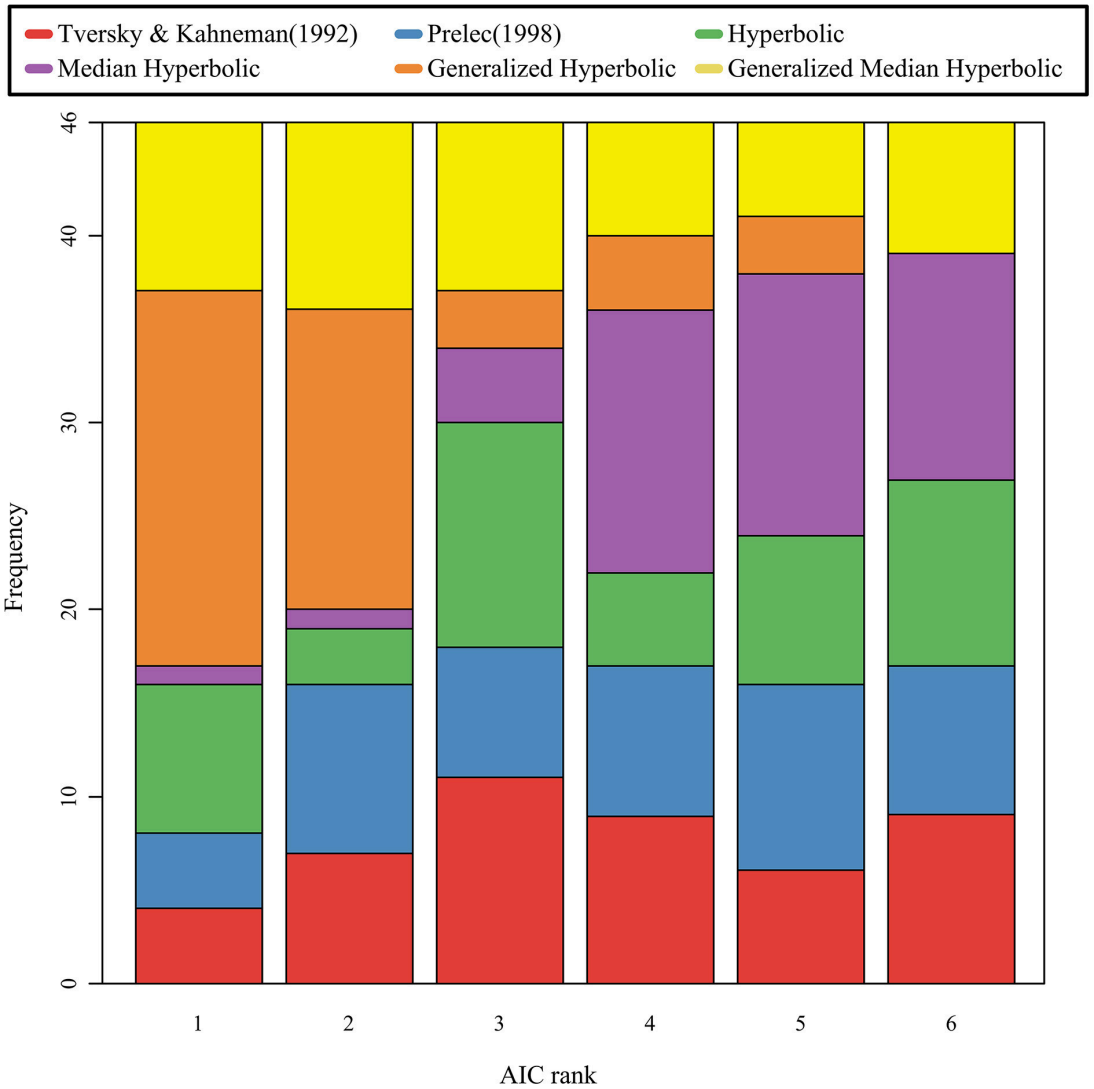
**The stacked bar chart of the AIC ranks for the six models**.

We used the same procedure as in the study by Gonzalez and Wu (1999) for estimating the parameters of both functions. Moreover, we computed the Akaike information criterion (AIC), which indicates the goodness of fit for the models. The cumulative distributions of the AIC values for the six models are shown in Figure 4; the stacked bar chart of the AIC ranks was shown in Figure 5. As shown in Figures 4, 5, the generalized hyperbolic time discounting model fitted better than the other models.

We also analyzed individual data, and computed AIC and BIC for each participant. Since patterns of AIC and BIC were similar for each participant data, we only analyzed the AIC values. We coded the rank of the AIC value of the model for each participant in a manner similar to that shown in Stott's (2006) study; we then conducted the Kruskal–Wallis test and the Mann–Whitney test using the Bonferroni correction to compare the median rank of one model with the median rank of another model for each combination of models. A Kruskal–Wallis test revealed a significant effect of models on the rank of the AIC value (χ^2^(5) = 59.3, *p* < 0.001). A *post hoc* test using Mann–Whitney tests with the Bonferroni correction suggested that the general hyperbolic model was significantly better than all five other models (*p* < 0.05), and that the median hyperbolic model was significantly worse than the general median hyperbolic model. There are no other significant effects, and the other four models tested show no significant differences among each other.

This result suggests that the generalized hyperbolic model (Prelec, 1998; Luce, 2001) fitted better than the other models, and implies that the projection invariance property holds in the probability weighting function. In Stott's (2006) study, the best model was the one-parameter Prelec model in terms of the AIC measure. Although he did not consider the median time discounting model because we have proposed this model in this study, he analyzed all other models. Stott (2006) examined all combinations of probability weighting functions, value functions, and choice functions using 96 samples. His result was slightly different from that obtained in our analysis. As Stott (2006) suggested, these estimations may be related to the size of the payoffs used in the gamble stimuli. Therefore, further empirical study will be needed to clarify the origin of this difference. In spite of the difference between both studies, the one-parameter Prelec model is considered to be a special case of the generalized hyperbolic model. In this sense, the generalized hyperbolic model proposed by Prelec (1998) is considered to fit well for the present study, as well as for Stott's study.

**Figure 6:**
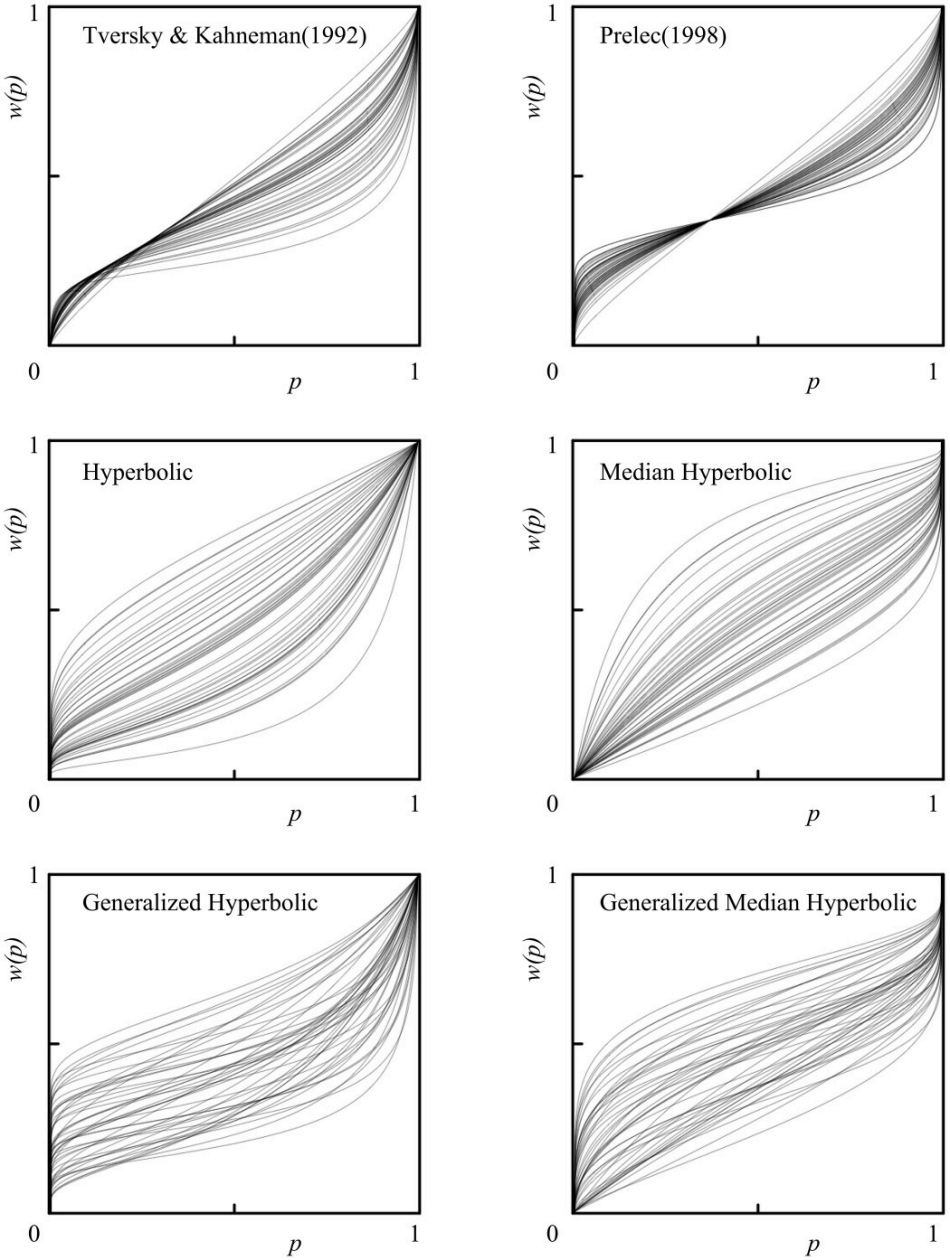
**Individual fits of all 46 participants in a single plot for each model**.

**Figure 7:**
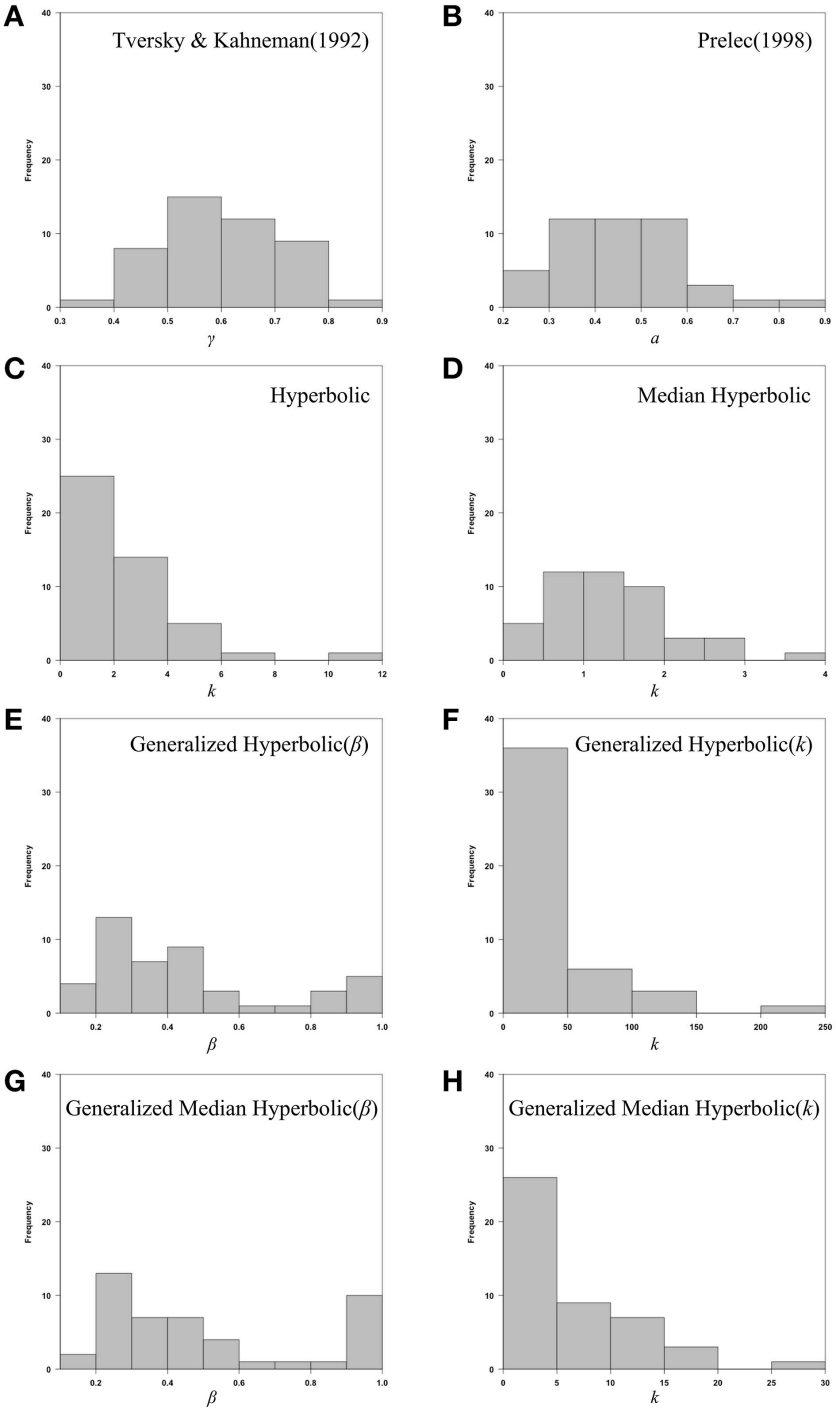
**Histograms of the estimated parameters of all six models across 46 participants: (A)** Tversky and Kahneman (1992) model(γ), (B) Prelec (1998) model (*a*), (C) Hyperbolic model (*k*), (D) Median Hyperbolic (*k*), (E) Generalized Hyperbolic(β), (F) Generalized Hyperbolic(*k*), (G) Generalized Median Hyperbolic(β), and (H) Generalized Median Hyperbolic(*k*).

**Figure 8:**
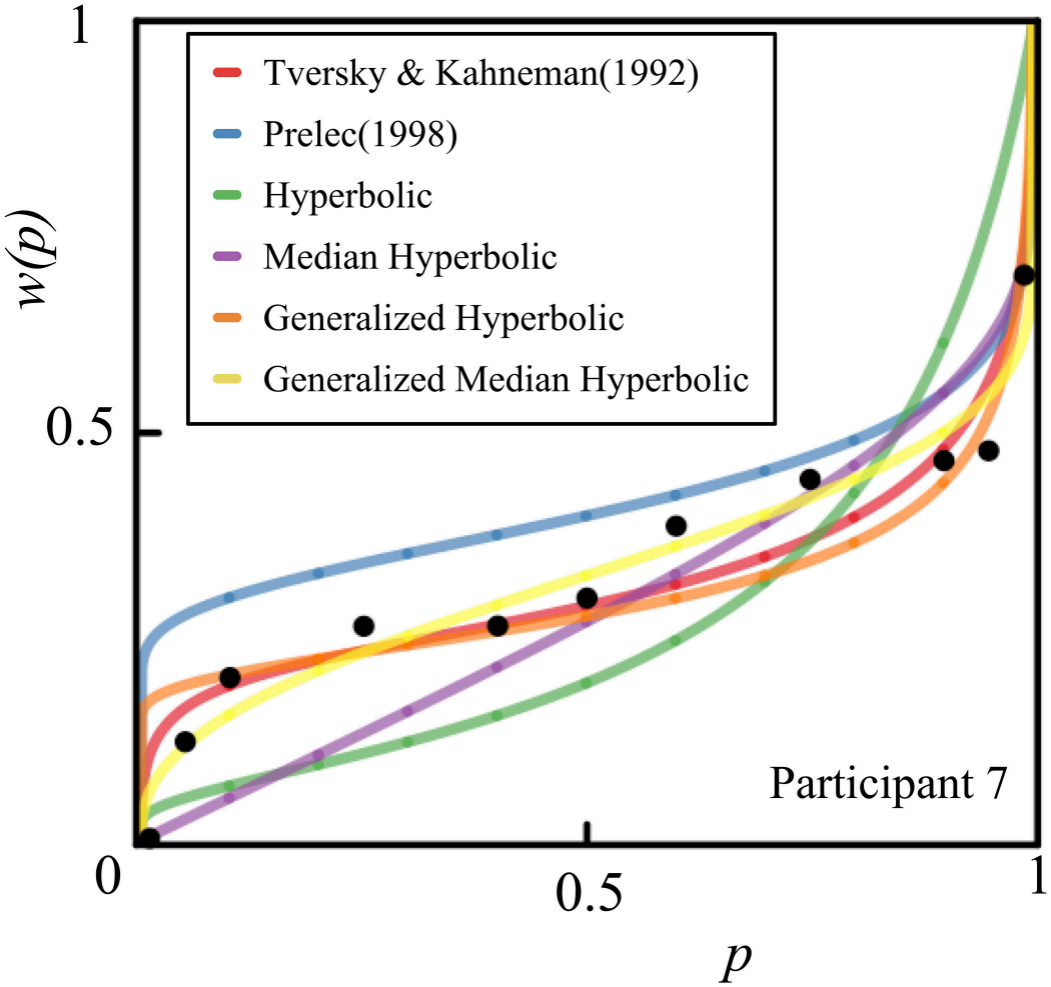
**The estimated probability weighting function of the outlier (Participant 7)**.

We also provide individual fits of all 46 participants in a single plot for each model of interest in Figure 6. Histograms of the estimated parameters of all six models across 46 participants are shown in Figure 7. In Figure 7, the following parameters were shown: (A) Tversky and Kahneman (1992) model(γ), (B) Prelec (1998) model (*a*), (C) Hyperbolic model (*k*), (D) Median Hyperbolic (*k*), (E) Generalized Hyperbolic(β), (F) Generalized Hyperbolic(*k*), (G) Generalized Median Hyperbolic(β), and (H) Generalized Median Hyperbolic(*k*). The best-fit model was the generalized hyperbolic model. However, there was only one outlier in the histogram of the parameter *k* for the generalized hyperbolic model, as shown in Figure 7. The estimated probability weighting function of the outlier (Participant 7) is shown in Figure 8. The best-fit model for Participant 7 was the generalized median hyperbolic model. In summary, through the individual analysis of the probability weighting function, we that conclude the general hyperbolic model tended to fit most of the data relatively better than the other models examined in this study.

## Conclusion

The present study proposed a probability weighting function derived from a hyperbolic time discounting model by assuming a geometric distribution. Moreover, our probability weighting function was derived from Loewenstein and Prelec's (1992) generalized hyperbolic time discounting model. The present study derived this hyperbolic-logarithmic model from the generalized hyperbolic time discounting model assuming Fechner's (1860) psychophysical law of time and a geometric distribution of trials. Since the geometric distribution is skewed, a logarithmic psychophysical function (−log *p*) was considered to be an approximation to the median of trials. Under this interpretation, the probability of the weighting function was derived from the generalized hyperbolic model using the median of geometrically distributed trials.

There are two primary contributions of this study. First, we derived the probability weighting function based on the generalized hyperbolic time discounting function. Second, we demonstrated the empirical study comparisons that fitted for six different probability weighting functions for 50 participants each corresponding to 165 unique gambles. This paper therefore provides theoretical and empirical support for a psychological interpretation of the probability weighting function from a time discounting perspective.

Further theoretical and empirical studies will be required to examine the shape of the probability weighting function. The results of the psychological experiment indicated that the expected value model of generalized hyperbolic discounting was a better fit than previous probability weighting decision-making models. However, we do not think that strong conclusions are ill advised owing to the limited number of participants, as compared to those in Stott's (2006) study. Moreover, the weighting function might vary in response to changes in psychological factors. For instance, Kusev et al. (2009) found that exaggerated risk was caused by the accessibility of events in memory. The results suggested that the weighting function varied as a function of the accessibility of events. This finding was supported by the studies of Kusev and van Schaik (2011) and Jones and Oaksford (2011), in which they applied the findings from Kusev et al.'s (2009) study on transactional content on the temporal and probabilistic discounting of costs. Since the limited sample size of this study is the same as that in previous studies (Tversky and Kahneman, 1992; Gonzalez and Wu, 1999), further experiments with larger sample sizes and manipulating psychological factors—such as the accessibility of events in memory (Kusev et al., 2009)—will be required to more fully examine the psychophysical model of probability weighting functions.

## Author contributions

KT supervised the project, and made mathematical models and wrote the manuscript. HM conducted the experiments and analyzed the data.

## Funding

This study was supported by JSPS Grant-in-Aid for Scientific Research (A), No.24243061.

### Conflict of interest statement

The authors declare that the research was conducted in the absence of any commercial or financial relationships that could be construed as a potential conflict of interest.
