# Supplementary figures and images for: Probability Weighting Functions Derived from Hyperbolic Time Discounting: Psychophysical Models and Their Individual Level Testing
Source: Front Psychol. 2016 May 26;7:778. doi: 10.3389/fpsyg.2016.00778 (PMC4881414; doi:10.3389/fpsyg.2016.00778)

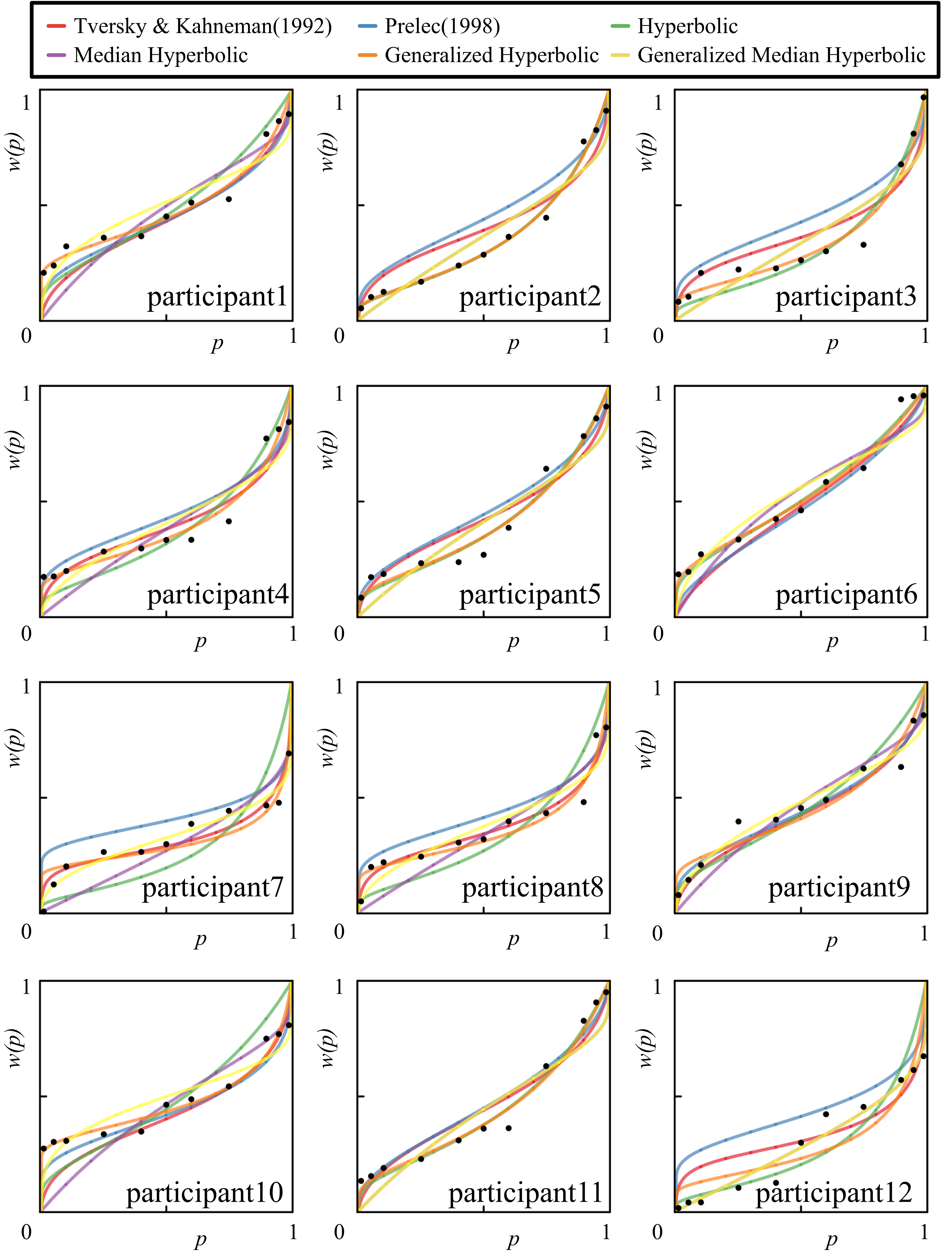

Supplement: Supplementary file 2 [file Image1.TIFF]

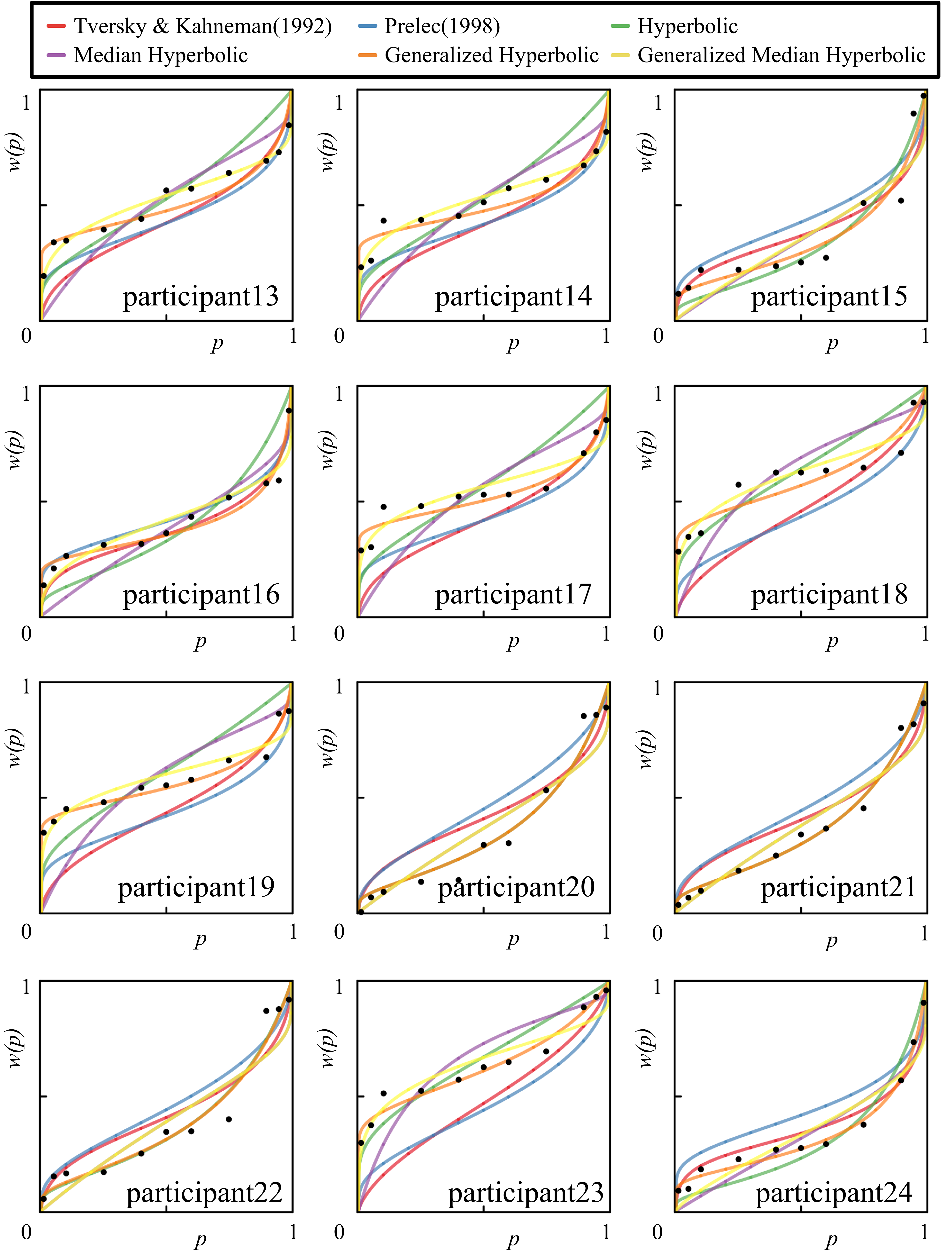

Supplement: Supplementary file 3 [file Image2.TIFF]

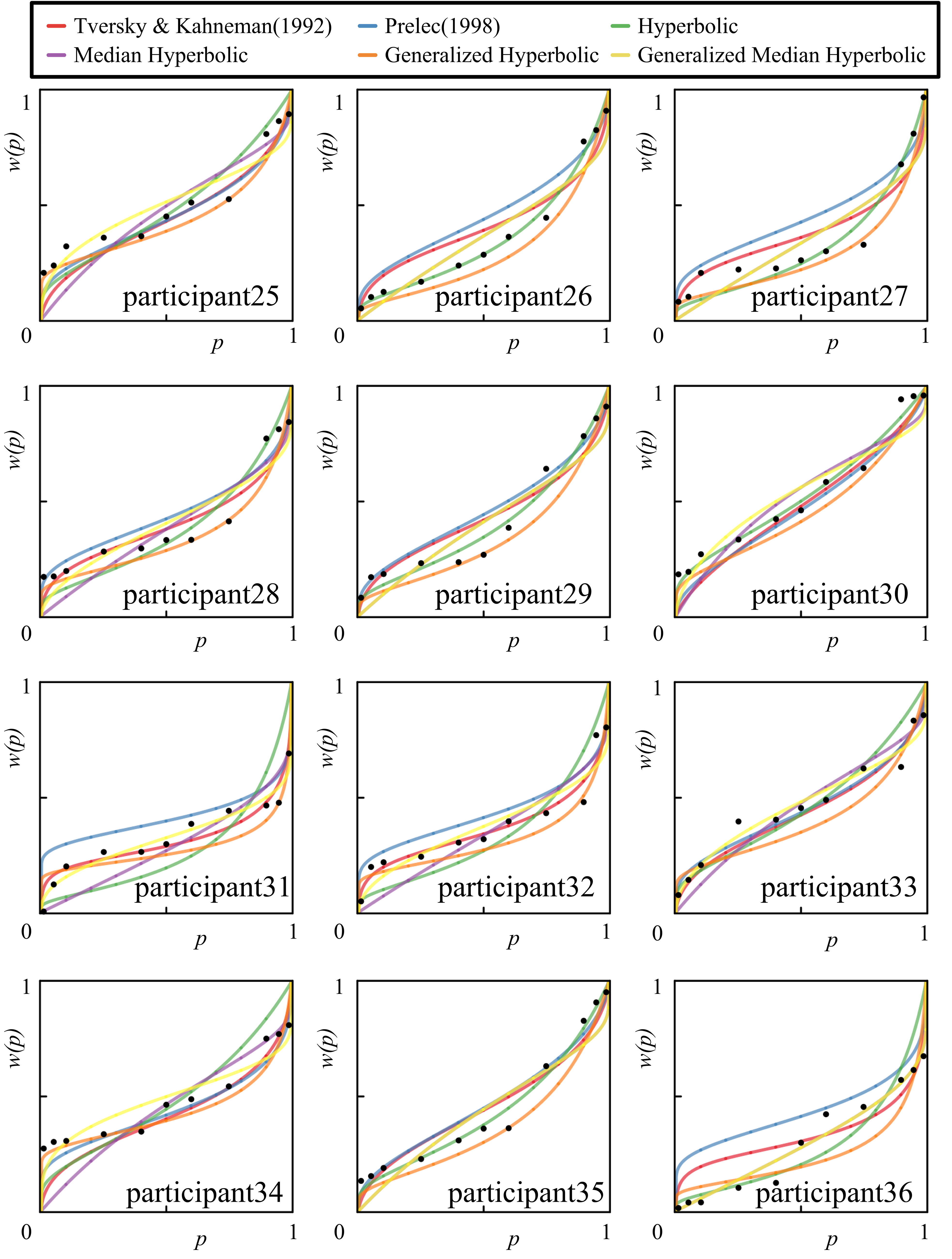

Supplement: Supplementary file 4 [file Image3.TIFF]

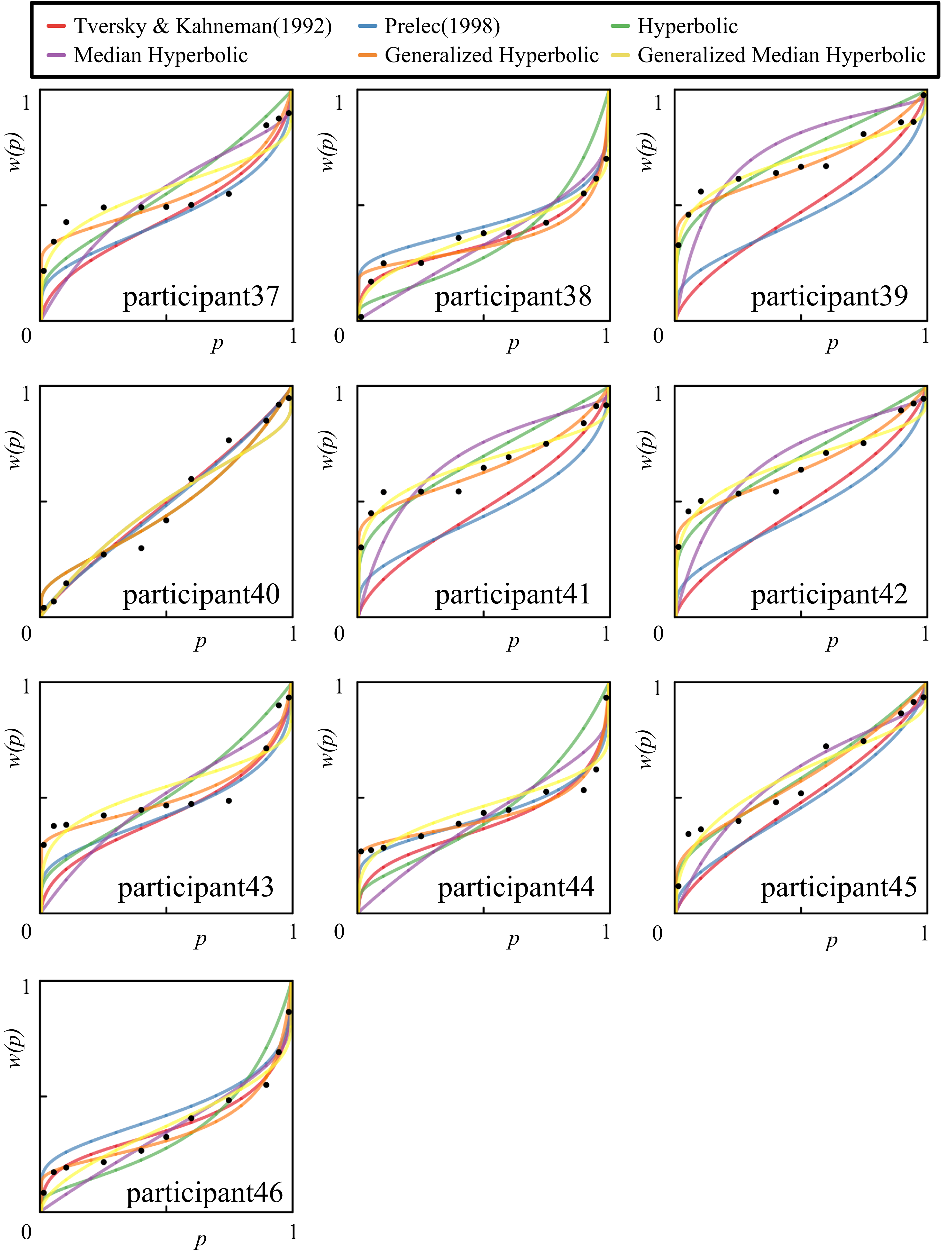

Supplement: Supplementary file 5 [file Image4.TIFF]
